# Do primary care professionals agree about progress with implementation of primary care teams: results from a cross sectional study

**DOI:** 10.1186/s12875-016-0541-9

**Published:** 2016-11-22

**Authors:** E. Tierney, M. O’Sullivan, L. Hickey, A. Hannigan, C. May, W. Cullen, N. Kennedy, L. Kineen, A. MacFarlane

**Affiliations:** 1 Graduate Entry Medical School, University of Limerick, Limerick, Ireland; 2 Faculty of Health Sciences, University of Southampton, Southampton, UK; 3 Urban General Practice, Health Sciences University College Dublin, Dublin, Ireland; 4 Faculty of Education & Health Sciences, University of Limerick, Limerick, Ireland; 5 Health Service Executive, Merlin Park, Regional Hospital, Galway, Ireland

**Keywords:** Policy implementation, Health care surveys, Primary health care, Patient care team, General practice, Interdisciplinary teams, Health policy

## Abstract

**Background:**

Primary care is the cornerstone of healthcare reform with policies across jurisdictions promoting interdisciplinary team working. The effective implementation of such health policies requires understanding the perspectives of all actors. However, there is a lack of research about health professionals’ views of this process. This study compares Primary Healthcare Professionals’ perceptions of the effectiveness of the Primary Care Strategy and Primary Care Team (PCT) implementation in Ireland.

**Methods:**

Design and Setting: e-survey of (1) General Practitioners (GPs) associated with a Graduate Medical School (*N* = 100) and (2) Primary Care Professionals in 3 of 4 Health Service Executive (HSE) regions (*N* = 2309). After piloting, snowball sampling was used to administer the survey. Descriptive analysis was carried out using SPSS. Ratings across groups were compared using non-parametric tests.

**Results:**

There were 569 responses. Response rates varied across disciplines (71 % for GPs, 22 % for other Primary Healthcare Professionals (PCPs). Respondents across all disciplines viewed interdisciplinary working as important. Respondents agreed on lack of progress of implementation of formal PCTs (median rating of 2, where 1 is no progress at all and 5 is complete implementation). GPs were more negative about the effectiveness of the Strategy to promote different disciplines to work together (median rating of 2 compared to 3 for clinical therapists and 3.5 for nurses, *P* = 0.001). Respondents identified resources and GP participation as most important for effective team working. Protected time for meetings and capacity to manage workload for meetings were rated as very important factors for effective team working by GPs, clinical therapists and nurses. A building for co-location of teams was rated as an important factor by nurses and clinical therapists though GPs rated it as less important. Payment to attend meetings and contractual arrangements were considered important factors by GPs but not by nurses or clinical therapists.

**Conclusion:**

PCPs and GPs agree there is limited PCT implementation. GPs are most negative about this implementation. There is some disagreement about which resources are most important for effective PCT working. These findings provide valuable data for clinicians and policy makers about implementation of interdisciplinary teams in primary care.

**Electronic supplementary material:**

The online version of this article (doi:10.1186/s12875-016-0541-9) contains supplementary material, which is available to authorized users.

## Background

The benefits of delivering primary care through interdisciplinary teams are well established [1–4]. Specific benefits have been reported for patients with diabetes [5], hypertension [6], obesity [7] and depression [8]. Heath Care Professionals have also noted advantages including improved professional satisfaction [9].

In some countries, such as the UK, interdisciplinary team work in primary care has been gradually normalized through organic processes over relatively long periods of time and is now routinely incorporated into healthcare system redesign. In other countries like the United States, the Patient-Centered Medical Home model is seen as a strategic opportunity to modernise primary care [10] and early demonstration projects show some promise. However, full implementation has been slow, falling behind other developed countries which have a commitment to primary care [10, 11]. Ireland is similar, where attempts are being made to implement ‘top down’ policies [12–16], aimed at encouraging the rapid development of interdisciplinary teamwork as a means of improving the quality and increasing the efficiency of primary care. These policy shifts are part of a response to the increasing demands placed on primary care by the major demographic and epidemiological transitions experienced by all of the advanced economies in recent years [17–19].

In Ireland, substantial reform of primary care was enshrined in policy in 2001 [16]. This Primary Care Strategy proposed an inter-disciplinary approach to primary care, based around Primary Care Teams (PCTs) [20]. PCTs would comprise a wide range of health professionals, located in a single primary care centre [16, 21]. Members of the PCT would include GPs, nurses/midwives, health care assistants, home helps, physiotherapists, occupational therapists, social workers and administrative personnel. A wider primary care network of other primary care professionals would support the team to provide services for the enrolled population of each primary care team. GPs would be encouraged to join together their existing lists of enrolled individuals and families with the PCTs.

The aims of the proposed developments were to provide: a greatly strengthened primary care system; an integrated, inter-disciplinary, high-quality, team-based and user-friendly set of services for the public; enhanced capacity for primary care to complement the existing diagnosis and treatment focus in the areas of prevention, early intervention, rehabilitation and personal social services (20] (page 13).

However, the implementation of this Strategy over the past decade has been described as ‘very challenging’ [22]. The limited evidence suggests that PCT working is not routine and it is still rare for GPs to work alongside other health professionals to provide an integrated primary care system [23]. Furthermore, the rates of adoption or adaption of actors involved has not been documented [24]. A key challenge for healthcare systems like Ireland’s is how best to deliver new interventions across the wide diversity of possible settings [18, 25, 26]. This poses important problems of *translational* research around the interactions of actors and interests through which policies are implemented [27, 28] and the role of policy and practice contexts in shaping barriers and facilitators to implementation [29, 30]. Understanding the process of implementing policy in this particular area is further complicated by a diversity of inter disciplinary team types and multiple definitions in use across settings [12, 13, 31–34]. In this study we adopted the term ‘interdisciplinary’ team working as this is the term used in the Irish Primary Care Strategy [16].

Using this policy intervention in Ireland as a vehicle, we want to address this translational problem by examining the ways that different professional groups understand and interpret experiences of interdisciplinary team working. Because the existing evidence tends to focus on the perspectives of specific professional groups [35–40], this is an area where surprisingly little is known [41]. This is problematic given the fact that the opinions of a variety of professionals, should be taken into account during implementation processes [41]. Following recommendations to use theory in translational research [29], we drew on Normalisation Process Theory (NPT) [12] to inform this research. NPT concentrates on the notion of normalisation in health care settings i.e., the point at which a new way of working becomes routine and taken-for-granted. It has four constructs coherence, cognitive participation, collective action and reflexive monitoring which allow exploration of *sense making, engagement, enactment and appraisal* of the practice or intervention in question.

The unique feature of NPT compared with other implementation theories is that it has developed from comprehensive analyses of the implementation of complex interventions in healthcare settings representing a good ‘fit’ with our interest in the implementation of PCTs as a complex intervention in the health service in Ireland. A recent review found that NPT is a beneficial heuristic device to explain and guide implementation processes and recommended that it be used with multiple stakeholders to enable analysis of implementation from a range of perspectives [42, 43].

The aim of this study was to better understand the perspectives on policy implementation of participating professional groups and to understand barriers to the implementation of interdisciplinary team working across disciplines.

## Methods

### Study context

The Health Service Executive (HSE) is a national publicly funded organisation which provides health and social services in Ireland. It is divided into four regions to deliver those services at regional level. These regions are HSE South, HSE West, HSE Mid-Leinster and HSE North East.

General Practitioners (GPs): There are about 2500 GPs in Ireland. Most are in private practice but also have contracts with the HSE to provide services to those eligible for publicly funded primary care.

Primary Care Teams in Ireland were initially implemented in 2001 after the publication of the Primary Care Strategy. The intended composition is General Practice staff (including GPs, practice nurses, practice managers) and HSE staff (including Public Health Nurses (PHN), Registered General Nurses (RGN), Physiotherapists, Occupational Therapists (OT), Speech and Language Therapists (SLT) Social Workers and Administrative staff) with additional support from wider primary care networks, including pharmacists, dieticians and other professionals.

### Participants

The participant groups in this study were staff in 100 general practices affiliated with a Graduate Entry Medical School in the Mid-West of Ireland. These practices represent the range of general practice types in the Irish context, the majority of which are based in city and/or town locations with GPs in full time employment and operating group practices [44].

Primary health care staff, employed by the HSE in three of Ireland’s four HSE regions were identified via the National Primary Care Office within the HSE. There are 2309 fulltime equivalent posts employed by the HSE in 380 PCTs in these regions, which serve a population of 3.5 million people.

### Survey design

The survey instrument developed by the research team consisted of 32 questions and comprised closed and open ended questions. The survey questions and content was designed following the principles for constructing web surveys [45–47] with reference to the Primary Care Strategy [16] and other pertinent literature on PCTs in Ireland [24, 48]. We reviewed the Primary Care Strategy and literature for key concepts and common findings about implementation of PCTs.

We also drew on our knowledge of Normalisation Process Theory [49] to formulate questions.

NPT informed questions were designed to explore the respondent’s views of implementation of the Primary Care Strategy in Ireland. Specifically, Coherence was explored by Q1 and 2 about perceived importance of PCT working – does it *make sense*?; Cognitive Participation by Q6 and 7 which related to *engagement* in the PCT; Collective Action by Q10 and 11 which asked about resources needed to *enact* PCT working; and Reflexive Monitoring by Q3, 8 and 9 which explored *appraisals* of progress with implementation of the Primary Care Strategy overall and the implementation of participants’ specific PCTs (see Additional file 1).

Respondents were also asked about the composition of their PCT and to give information on their own background (including demographic information, qualifications, number of years’ experience). Following best practice [50, 51], the survey was piloted with relevant health professionals. This helped us to clarify goals and identify practical problems [50, 52]. Issues of flow, salience and administrative ease were identified [52]. Self-reported completion time for those who finished the survey was on average 15 min, consistent with studies reported elsewhere [53].

The procedure for sending out the survey followed an adapted Dillman Tailored Design Method (TDM) to maximise response rates. The Dillman TDM consists of a series of precisely laid out steps [54, 55] for example sending an advance notice about the forthcoming study, and follow up reminder emails sent at regular intervals [55–58].

In addition, the questionnaire was delivered electronically, had a clear focus, was concise and clearly designed, with a simple layout. A researcher was available to answer any queries [50, 51, 59].

The survey was piloted and conducted over a four month period in 2014.

### Sampling method

The survey was sent by email to all GP Practices (*n* = 100) affiliated with the Graduate Entry Medical School. No mailing list of all primary healthcare staff employed by the HSE in the three regions existed so a snowball sampling method was used: HSE managers and Primary Care Transformation Development Officers (*n* = 39) in the three HSE regions were sent the survey by email and asked to distribute to all relevant staff.

### Statistical methods

Descriptive statistics are presented as count (percentage) for categorical data and median (first quartile, third quartile) for rating scales and rankings. Cronbach’s alpha was used as a measure of internal consistency of items related to progress of implementation and effectiveness of strategy and team working. Non-parametric tests were used to compare medians across groups. A 5 % level of significance was used for all statistical tests. SPSS version 21 for Windows was used for the analysis.

## Results

### Response rate

There were 569 eligible responses including 71 GPs (response rate of 71 %) and 498 other healthcare professionals (response rate at most 22 % of HSE full-time equivalent posts). The distribution of occupations within the HSE regions and within the sample is given in Table 1.

**Table 1:** Distribution of occupations in the HSE regions and the survey sample

Occupation	% employed by HSE^a^	%o f valid responses^b^
Public health and general registered nurse	55.2 %	17.7 %
Physiotherapist	12.4 %	17.7 %
Occupational therapist	10.6 %	21.6 %
Speech and language therapist	8.1 %	13.8 %
Social worker	2.0 %	3.7 %
Dietician	1.7 %	5.0 %
Psychologists/Counsellors	1.6 %	3.4 %
Other	8.4 %	17.1 %

^a^Full-time equivalent posts

^b^Survey respondents who gave their occupation excluding general practitioners

Respondents from the HSE in the main comprised OTs, Physiotherapists and SLTs (hereafter and for the purposes of this paper grouped together and called clinical therapists). While most occupations within the HSE were adequately represented in the sample, nurses were under represented. Of the 71 GPs who responded, 34 % were in rural practices, 41 % were in mixed urban/rural practices and 24 % were in urban practices, largely representative of all GP practices in Ireland. Response rates across the three regions were broadly similar (Table 2).

**Table 2:** Demographics of respondents (*n* = 569)

Characteristic	*n*	% of total responses	% of valid responses^a^
Age group			
≤ 35	106	18.6	25.1
36–49	197	34.6	46.7
≥ 50	119	20.9	28.2
Not given	147	25.8	
Gender			
Female	344	60.5	81.5
Male	78	13.7	18.5
Not given	147	25.8	
Occupation			
Occupational therapist	77	13.5	18.0
General practitioner	71	12.5	16.6
Physiotherapist	63	11.1	14.8
Public health/registered general nurse	63	11.1	14.8
Speech and language therapist	49	8.6	11.5
Manager	25	4.4	5.9
Dietician	18	3.2	4.2
Social Worker	13	2.3	3.0
Psychologist/Counsellor	12	2.1	2.8
Other occupations^b^	36	6.3	8.4
No occupation given	142	25.0	
Years since qualification			
1–5	32	5.6	7.7
5–10	76	13.4	18.3
10–15	89	15.6	21.4
15+	219	38.5	52.6
Not given	153	26.9	
HSE Region			
HSE West	174	30.6	42.2
HSE Dublin Mid-Leinster	143	25.1	34.7
HSE South	95	16.7	23.1
Not given	157	27.6	
Member of a formal primary care team			
Yes	388	68.2	78.1
No	109	19.2	21.9
Not applicable/not given	72	12.6	

^a^% of responses excluding not given

^b^home help, community pharmacist, community worker, dentist, primary care facilitator, community doctor, general practice administration staff, general practice nurse, community welfare officer, area medical officer

### Demographics

Of those who provided valid demographic information (*n* = 427), respondents were predominantly female (82 %) and the majority (72 %) were aged less than 50 years.

The majority of respondents (53 %) were 15 years or more post qualification.

Of the 71 GPs, the majority (62 %) were male; aged 50 years or more (57 %) and were 15 years or more post qualification (67 %), representative of the GP profile in Ireland [44].

### Composition of primary care teams

78 % of respondents reported that they were a member of a formal PCT. Of those who were a member of a team (*n* = 388), 34 % were a member of two or more teams and 81 % had been a member of a team for five years or less.

72 % reported that they frequently or very frequently attended PCT meetings. Only 7 % reported that they never attended meetings. When asked to name who was on their PCT, the most frequently cited profession was PHN (77 %), followed by OT (75 %), Physiotherapist (75 %), GP (57 %), SLT (52 %) and RGN (51 %). Pharmacists (3 %), Community Welfare Officers (6.7 %) and Social Workers (9.5 %) were the least frequently cited professions.

### Perceptions of PCT working and progress with PCT implementation in Ireland

Respondents rated *the importance of interdisciplinary working* as 5, on average; on a 5 point scale where 1 is not at all important and 5 is extremely important (Table 3). Comparing the three largest groups (clinical therapists, GPs and nurses), while both nurses and clinical therapists rated the importance of interdisciplinary working higher on average compared to GPs (median of 5 compared to 4 for GPs, *P* <0.001), all three groups rated it as important.

**Table 3:** Characteristics of primary care teams for named members of formal PCTs (*n* = 388)^a^

Characteristic	*n* (%)
How many teams are you a member of?	
One	249 (65.7)
Two	84 (22.2)
Three or more	46 (12.1)
How long have you been a member of the team?	
0–1 year	70 (19.2)
1–5 years	225 (61.8)
5 or more years	69 (19.0)
How often do you attend meetings?	
Very frequently	172 (44.8)
Frequently	105 (27.3)
Infrequently/Rarely/Never	107 (27.9)
Who is on your team?	
Public health nurse	300 (77.3)
Occupational therapist	293 (75.5)
Physiotherapist	291 (75.0)
General practitioner	221 (57.0)
Speech and language therapist	202 (52.1)
Registered general nurse	199 (51.3)
Home help co-ordinator	176 (45.4)
Dietician	136 (35.1)
Administrator	128 (33.0)
Clinical psychologist	83 (21.4)
Counsellor	46 (11.9)
Community representative	40 (10.3)
Social worker	37 (9.5)
Community welfare officer	26 (6.7)
Pharmacist	10 (2.6)
Other^b^	28 (7.2)

^a^Missing responses for some characteristics - % of valid responses reported

^b^Community Psychiatry/Mental Health, Community Worker, Drugs and Alcohol Counsellor, Chiropodist, Elder Day Care Managers, Care Provider Agency, Community Hospital Representative, Hospital Palliative Nurse, Diabetic Nurse, Smoking Cessation Officer, Specialist Liaison Nurse

The following four items on the questionnaire (Q2, Q3, Q8, Q9) related to progress with implementation and effectiveness of the Primary Care Strategy and team working. The value of Cronbach’s alpha was 0.7 for these items indicating acceptable internal consistency.

Respondents rated the *general progress of implementation* of formal PCTs as 2, on average, on a five point scale where 1 is no progress at all and 5 is complete implementation (Table 3). 32 respondents (6 %) reported no progress at all and 4 (1 %) reported complete implementation. Comparing the three largest groups, clinical therapists tended to have more positive views on progress (median of 3 compared to 2 for both GPs and nurses, *P* <0.001).

Views on the *effectiveness of the Primary Care Strategy* to promote different disciplines to work together were slightly more positive with a rating across all respondents of 3, on average; on a five point scale where 1 is not at all effective and 5 is extremely effective. Again, GPs were more negative about the effectiveness of the Primary Care Strategy to promote interdisciplinary working with an average rating of 2 compared to 3 for clinical therapists and 3.5 for nurses (*P* = 0.001) (Table 4).

**Table 4:** Health professional views of policy implementation; Median rating (1st quartile, 3rd quartile) for all respondents (*n* = 569) and by occupation

Occupation	Importance of multidisciplinary work (1 = not at all important, 5 = extremely important)	General progress of implementation (1 = no progress at all, 5 = complete implementation)	Effectiveness of HSE strategy (1 = not at all effective, 5 = extremely effective)
Clinical Therapist (*n* = 189)	5 (5, 5)	3 (2, 3)	3 (2, 4)
GP (*n* = 71)	4 (3, 5)	2 (2, 2)	2 (1, 3)
Nurse (*n* = 63)	5 (5, 5)	2 (2, 3)	3.5 (3, 4)
Manager (*n* = 25)	5 (5, 5)	3 (2, 3)	3 (3, 4)
Social worker/Psychologist/Counsellor (*n* = 25)	5 (4, 5)	2 (2, 3)	3 (2, 4)
Dietician (*n* = 18)	5 (5, 5)	3 (2, 3)	3 (2, 4)
Other occupations (*n* = 36)	5 (4, 5)	2 (2, 3)	3 (2, 4)
No occupation given (*n* = 142)	5 (4, 5)	3 (2, 3)	3 (3, 4)
All respondents (*n* = 569)	5 (4, 5)	2 (2, 3)	3 (2, 4)

Views on the progress of *implementation of the primary care teams which respondents were members of* (*n* = 388) were also slightly more positive than the views on general progress with an average rating of 3 on a 5 point scale where 1 is no progress at all and 5 is complete implementation. GPs tended to have more negative views about the teams which they were members of than all other respondents (median of 2 compared to 3 for all others) though this difference was not significant (*P* = 0.08).

The *effectiveness of the team working together* was rated by team members as 3 on average on a five point scale where 1 is not at all effectively and 5 is very effectively. Comparing the three largest groups, both nurses and GPs had more negative views on the effectiveness of the team working together compared to clinical therapists (median of 2 compared to 3, *P* = 0.01) (Table 5).

**Table 5:** Views on the progress of implementation of primary care teams by members of those teams Median rating (1st quartile, 3rd quartile) for all named members of formal PCTs (*n* = 388) and by occupation

Occupation	Progress of implementation of PCTs you are a part of (1 = no progress at all, 5 = complete implementation)	Effectiveness of PCT members working together as a formal team (1 = not at all effectively, 5 = very effectively)
Clinical Therapist (*n* = 148)	3 (2, 4)	3 (2, 4)
GP (*n* = 61)	2 (1.5, 3)	2 (2, 3)
Nurse (*n* = 51)	3 (2, 3)	2 (2, 3)
Social worker/Psychologist/ Counsellor (*n* = 16)	3 (2.5, 4)	3 (2, 4)
Manager (*n* = 7)	3 (2, 4)	2 (2, 3.5)
Dietician (*n* = 12)	3 (2, 3.5)	2 (2, 3)
Other occupations (*n* = 21)	3 (2, 4)	3 (3, 4)
No occupation given (*n* = 72)	3 (2, 4)	3 (2, 4)
All team members (*n* = 388)	3 (2, 4)	3 (2, 4)

### Requirements for effective PCT working to support its implementation

Respondents ranked resources and GP participation as most important factors to promote effective team working with community participation and waiting list systems ranked as least important factors. These findings were consistent across the three largest groups (Table 6).

**Table 6:** Rank order of factors required for effective PCT working- views across three largest disciplines

Occupation	Most important factors^a^	Less important factors^b^
Clinical Therapist (*n* = 189)	Resources GP participation Communication Leadership	Time of meetings Clarity re roles Skills, knowledge and training Community participation Waiting list system
GP (*n* = 71)	Resources Time of meetings GP participation Leadership	Clarity re roles Skills, knowledge and training Communication Community participation Waiting list system
Nurse (*n* = 63)	GP participation Resources Time of meetings Leadership	Clarity re roles Skills, knowledge and training Communication Community participation Waiting list system

^a^Median ranking of importance 1–4 on a 9 point scale where 1 is most important and 9 is least important

^b^Median ranking of importance of 5 or above on a 9 point scale where 1 is most important and 9 is least important

When asked about *the importance of resources for PCT meetings*, protected time for meetings and capacity to manage workload associated with meetings were rated as very important by the three largest groups. A building for co-location of teams was rated as important by nurses and clinical therapists though GPs rated it as less important. Payment to attend meetings and contractual arrangements were considered important resources by GPs but not by nurses or clinical therapists (Table 7).

**Table 7:** Ranking of required resources for effective team working by three largest groups

Occupation	Most important resources^a^	Less important resources^b^
Clinical Therapist (*n* = 189)	Capacity to manage workload associated with meetings Protected time for meetings PCT building to have co-located team members	Payment for attending meetings Contractual arrangements
GP (*n* = 71)	Capacity to manage workload associated with meetings Protected time for meetings Payment for attending meetings Contractual arrangements	PCT building to have co-located team members
Nurse (*n* = 63)	Capacity to manage workload associated with meetings Protected time for meetings PCT building to have co-located team members	Payment for attending meetings Contractual arrangements

^a^Median rating of importance above 3 on a five point scale where 1 is not at all important and 5 is very important

^b^Median rating of importance of 3 or below on a five point scale where 1 is not at all important and 5 is very important

## Discussion

### Summary

The majority of respondents in this study reported little progress or no progress at all with implementation of the Primary Care Strategy in Ireland in general. Clinical therapists were more positive about PCT implementation than nurses or GPs. GPs were most negative about implementation of the specific PCTs that they have experience of.

Resources and GP participation were considered important factors to promote team working across all disciplines. Payment for meetings and contractual arrangements were considered more important resources for effective team working for GPs than for other professions. Working from a co-located PCT building was considered less important by GPs than other professions.

### Strengths and limitations

The majority of the sample were named members of a formal PCT, providing us with the views of experienced professionals working across established inter disciplinary teams in Ireland. Over a quarter of these, however, did not frequently attend meetings giving us an insight on implementation from those with different levels of engagement as recommended by Carlfjord and Festin [41].

There are many hurdles to accessing the many different actors involved in the policy process [60]. The variation in response rate from 71 % for GPs to 22 % for HSE staff should be viewed in the context of weaknesses in health information systems in the HSE – there is no mailing list of all HSE staff in the three regions. We were dependent on HSE managers and Transformation Development Officers to distribute the survey to relevant staff but had no way of knowing how many of these actually received it. While public health nurses (PHNs) and Registered General Nurses (RGNs) make up over half of the full-time equivalent posts in the HSE, only 18 % of the sample who gave an occupation were nurses. This underrepresentation may be due to the setting in which PHNs work in Ireland with limited access to email. We acknowledge this limitation and recommend that in future surveys, strategies to target a higher response rate across nursing professions be identified. Where a mailing list existed (GPs), responses were received from 71 of the 100 practices, despite GPs being recognized as a professional group from which it is difficult to obtain high response rates [59, 61, 62].

### Comparison with existing literature

It is known that health policy implementation must be informed by an understanding of the actors through which policies are developed and implemented [18, 27–29].

This study focused on the views of health professionals as key actors in the policy implementation process. Findings show that there is disagreement in Irish health professionals’ views about how effective a top down policy is to promote interdisciplinary working. GPs were more negative about the implementation of the Primary Care Strategy in relation to their specific PCTs than nurses and clinical therapists. The findings resonate with previous research in Ireland with single professional groups [63, 64] but, for the first time, show how professionals’ views compare with each other.

As reported elsewhere [24, 33, 38, 65] resources were considered important for PCTs to work effectively as was GP participation [38, 48, 64]. Consistent with other research [4, 24, 33, 40, 65] protected time for meetings and capacity to manage workload associated with meetings was rated as very important by nurses, clinical therapists and GPs.

However our findings show variation between the groups about the resources important for effective PCT implementation. Similar to previous research [66], payment to attend meetings and contractual arrangements were only rated important by GPs. This is likely to be explained by the self-employed nature of GPs’ fee-for-service contractual arrangements compared to the salaried positions of HSE staff in Ireland. This reflects findings in New Zealand where in salaried practices, doctors and nurses alike were employees and were particularly supportive of team working [33].

Interestingly, a PCT co-located building was rated as important by clinical therapists and nurses but not by GPs despite the evidence that co-location of practitioners may improve access to services and equipment that aid chronic disease management [19]. These findings are likely to be explained by the nature of the environment in which GPs work in Ireland –the majority work in privately owned facilities and may have discomfort about working in buildings owned by the HSE. We are currently exploring further in a qualitative study how these differential views impact on GP engagement and the nature of collaborations and dissidence between GPs and other professions [67–70].

Interestingly, there was agreement across GPs, clinical therapists and nurses that community participation and waiting list systems were the least important factors for effective PCT working. The former is strongly advocated in international and national policy but only 10 % reported that there was community participation on their PCT. The latter remains a significant challenge in the Irish healthcare service and so it is surprising that it would be considered least important by all disciplines.

### Implications for research and/or practice

In this study NPT was helpful to generate questions to explore views about implementation of a top –down policy with regard to patterns of shared and differential experiences across team members as well as the resources and factors important for implementation. We are limited in our ability to analyse findings *in depth* using NPT given the nature of survey data. However, we can identify questions for further research to generate evidence about the extent to which top-down initiatives are effective in general as mechanisms of translation. It would be valuable to explore how the constructs of NPT may impact one another in the implementation process. For example:

Does the ‘work’ involved in enacting and embedding PCT work (collective action) make more sense (coherence) to different types of professionals because of their relationship with the HSE and, consequently, directives for interdisciplinary working (cognitive participation)? Does PCT working simply sit better with HSE employees compared with GPs because of how they understand their ‘job’?

We found differences in opinion about co-location between GPs and other health professionals and it would be interesting to explore if co-location supports the flow of communication between professional groups (greater collective action), and why this may not be the same for GPs.

Does frequent involvement in PCT meetings (cognitive participation) mean that interdisciplinary working makes more sense (coherence) and thus, fuel efforts to reconfigure practices to overcome any barriers to interdisciplinary working (reflexive monitoring)?

We are currently exploring these issues in the aforementioned follow up qualitative study using NPT as our guiding framework.

## Conclusion

Primary Health Care Professionals in Ireland agree about the lack of progress with the implementation of PCTs. GPs are more negative than their colleagues from nursing and clinical therapy backgrounds. GPs also have different views about which resources are required to promote team working and these reflect health system funding and organisational factors. Attention to such differential views on PCT working is required to enhance the development and function of PCTs in Ireland.

## Endnotes

^1^ HSE. Staffing in Primary Care Teams in Ireland; Summary report: HSE 2014. Received via personal email correspondance.

## Additional file

Additional file 1: Primary Care Reform In Ireland Survey. (PDF 284 kb)

## Abbreviations

GPs: General practitioners
HSE: The Health Service Executive
PCTs: Primary care teams
PHN: Public health nurses
RGN: Registered general nurses
TDOs: Transformation development officers
