# Supplementary material for: Do primary care professionals agree about progress with implementation of primary care teams: results from a cross sectional study
Source: BMC Fam Pract. 2016 Nov 22;17:163. doi: 10.1186/s12875-016-0541-9 (PMC5120534; doi:10.1186/s12875-016-0541-9)
Supplement: Additional file 1: — Primary Care Reform In Ireland Survey. (PDF 284 kb) [file 12875_2016_541_MOESM1_ESM.pdf]

Intro page

There have been major changes in primary care over the last decade. Some are driven by the 2001 HSE Strategy for Primary Care, for example the implementation of formal Primary Care Teams. Others are driven by the healthcare providers working together 'on the ground' formally or informally.

This survey is designed to explore your

1. perceptions of progress with the implementation of formal Primary Care Teams (PCT)
2. knowledge of, and involvement in other interdisciplinary work between professionals in primary care

Section 1: Working with professionals from other disciplines

1. We know not everyone rates interdisciplinary working as important. How important is it for you to work with other primary care professionals?
1= not at all important and 5= extremely important

not at all important				extremely important
○	○	○	○	○

2. Do you think the HSE strategy on Primary Care A New Direction 2001 is an effective way to promote healthcare providers in primary care from different disciplines to work together?
1= not at all effective and 5= extremely effective

not at all effective				extremely effective
○	○	○	○	○

3. How would you perceive the general progress of implementation of formal PCTs <u>in Ireland</u> since 2001?
1= no progress at all and 5= complete implementation

no progress at all				complete implementation
○	○	○	○	○

Section 2 - Formal Primary Care Teams

Some people are members of one primary care team and some are members of more than one. Think of the PCT(s) that you are a member of and answer the questions below.

4. Are you a named member of a formal Primary Care Team?

- Yes
- No
- Not Applicable

5. How many formal Primary Care Teams are you a named member of?
Please type in number or not applicable

6. How long have you been a named member of the PCT(s)?
(Please type in a number or not applicable)

PCT 1

PCT 2

PCT 3

PCT 4

PCT 5

PCT 6

Not Applicable

7. Overall, how would you describe your attendance at formal PCT meetings? (We mean any meeting that you attend as a member of the formal primary care team i.e., clinical team meetings or business team meetings as applicable to you)

- Very Frequent
- Frequent
- Infrequent
- Rarely
- Never
- Not Applicable

8. Overall, how do you perceive the progress of implementation of the formal PCT(s) that you are part of?

no progress at all				complete implementation
○	○	○	○	○

9. Overall, do you think that PCT members are working effectively together as a formal team?

very effectively				not at all effectively
○	○	○	○	○

10. Overall, how important are the following resources to promote team members to work together effectively as a formal team ?
1= not at all important and 5= very important

	not at all important				very important
Protected time for meetings	○	○	○	○	○
Capacity to manage workload associated with meetings	○	○	○	○	○
Payment for attending meetings	○	○	○	○	○
Contractual arrangements (PCT working specified in contracts with HSE)	○	○	○	○	○
PCT Building to have co-located team members	○	○	○	○	○
Other	○	○	○	○	○
Other (please specify)	○	○	○	○	○

11. Overall, how important are the following factors to promote team members to work effectively together as a formal team?
Please rank in order of importance 1-9 (where 1= most important and 9= least important).

- [ ] Resources (a combination of Q10 above)
- [ ] Time of meetings
- [ ] GP participation
- [ ] Leadership
- [ ] Clarity regarding roles in PCTs
- [ ] Skills knowledge and training for PCT working
- [ ] Communication
- [ ] Community participation
- [ ] Waiting list system

12. Some people have described the following benefits from formal primary care team working. Have you experienced any of the following in your PCT(s)?
1= Have never experienced and 3= Have frequently experienced

	Have never experienced	Some experience	Have frequently experienced
Helps me to more effectively address the needs of patients with complex problems	○	○	○
Makes me feel less professionally isolated	○	○	○
Benefits the health of our local community	○	○	○
Improves patient health outcomes	○	○	○
Improves patient social outcomes	○	○	○
Improves patient psychological outcomes	○	○	○
It makes no difference	○	○	○
Other outcomes - please specify	○	○	○

Other (please specify)

13. Overall, what aspects of formal PCT working are most satisfying for you?

14. Overall, what aspects of formal PCT working are most challenging for you?

Section 3: Other forms of Interdisciplinary Working

Some people working in primary care have told us that there are other forms of interdisciplinary working that are not always captured by the HSE documentation of formal primary care team working.

These are based on formal meetings but, also, more informal meetings and conversations between professionals in primary care during, for example, coffees and lunches.

15. Do you agree with this statement?

strongly agree				strongly disagree
○	○	○	○	○

16. If you agree, is this kind of interdisciplinary work to (please tick):

☐ progress individual patient care e.g., to coordinate community care on hospital discharge, to refer a patient from one hospital to another?

☐ develop new services to improve care for a whole group of patients e.g., development of a new referral system, or a new therapeutic service?

Other (please specify)

17. If you have ticked any of the options in the previous question can you provide brief examples below.

Please include as many examples as you wish

18. What aspects of these formal and informal interactions across disciplines are most satisfying for you?

19. What aspects of these formal and informal interactions across disciplines are most challenging for you?

About You

Demographics

20. About your Professional Practice: Are you

- ☐ Counsellor
- ☐ Community Welfare Officer
- ☐ Psychologist
- ☐ Speech and language therapist
- ☐ Occupational therapist
- ☐ Nurse Practitioner PHN
- ☐ Nurse Practitioner RGN
- ☐ Practice Nurse
- ☐ Physiotherapist
- ☐ Home Help
- ☐ HSE manager
- ☐ GP
- ☐ Practice administrator
- ☐ Practice manager
- ☐ Transformation Development Officer
- ☐ PC Manager

Other (please specify)

21. Age

- <35
- 36-49
- >50

22. Gender

- Male
- Female

23. Years since finished professional training

- 1 - 5
- 5 - 10
- 10 - 15
- 15+

24. In which HSE area is your practice located

- ○ HSE Dublin Mid Leinster
- ○ HSE West
- ○ HSE South

25. County of practice:

26. Type of practice:

- ○ Rural
- ○ Urban
- ○ Mixed

27. Details of general practice (if applicable)

- ☐ Private
- ☐ Mixed
- ☐ GMS
- ☐ Not applicable

Section 3: About You

28. GMS List size if applicable:

- ≤ 500
- 500 999
- 1000 1499
- ≥ 1500
- Not applicable

29. Who is involved in your Primary Care Team? tick all that apply

- [ ] Clinical Psychologist
- [ ] Speech and Language Therapist
- [ ] Physiotherapist
- [ ] Occupational Therapist
- [ ] Registered General Nurse
- [ ] Public Health Nurse
- [ ] Dietician
- [ ] Pharmacist
- [ ] Administrator
- [ ] Home Help Coordinator
- [ ] Community Welfare Administration
- [ ] Counsellor
- [ ] GP
- [ ] Community Representative

Other (please specify)

Section 3: About You

30. Finally, is there anything else you would like to add about the issues raised in this survey?

31. We are interested in interviewing people about their experiences of the issues in this survey. Are you interested in becoming more involved in the next stage of this research project?

- Yes
- No

32. If yes, which of the following would you be willing to discuss further? You may tick more than one

- Good experience of formal PCT working
- Poor experience of formal PCT working
- Good experience of other forms of interdisciplinary working between professionals in primary care
- Poor experience of other forms of interdisciplinary working between professionals in primary care
- Other

Other (please specify)

If yes, please email us by selecting the **Email Me** link in the space below and we will get in touch.

Email Me

33. Sincere thanks for taking the time to complete this questionnaire. A donation will be made to a national charity on behalf of all participants who complete this survey. Please nominate your charity of choice in the box below. All nominations will be entered into a draw.

Final Page

For any enquiries contact:
Prof Anne MacFarlane,
Professor of Primary Healthcare Research
Graduate Entry Medical School
University of Limerick
Email: anne.macfarlane@ul.ie
